# Tamoxifen exposure and risk of oesophageal and gastric adenocarcinoma: a population-based cohort study of breast cancer patients in Sweden

**DOI:** 10.1038/sj.bjc.6603214

**Published:** 2006-06-06

**Authors:** E Chandanos, M Lindblad, C Jia, C A Rubio, W Ye, J Lagergren

**Affiliations:** 1 Unit of Esophageal and Gastric Research, Department of Molecular Medicine and Surgery, Karolinska Institutet, SE-171 76 Stockholm, Sweden; 2 Department of Medical Epidemiology and Biostatistics, Karolinska Institutet, SE-171 77 Stockholm, Sweden; 3 Department of Pathology, Karolinska University Hospital, SE-171 76 Stockholm, Sweden

**Keywords:** tamoxifen, oesophagus, gastric, adenocarcinoma, epidemiology

## Abstract

In a population-based cohort study of all women aged over 50 years with breast cancer in the Swedish Cancer Register in 1961–2003, those diagnosed before 31 December 1987 were regarded as unexposed to tamoxifen, whereas those diagnosed after that date were considered potentially exposed. Crosslinkages within the Cancer Register and the Registers of Death and Emigration enabled follow-up. Standardised incidence ratios (SIRs) of oesophageal and gastric cancer represented relative risks. Among 138 885 cohort members contributing with 1 075 724 person-years of follow-up, we found a nonsignificantly increased risk of oesophageal adenocarcinoma during the potential tamoxifen exposure period (SIR 1.60, 95% confidence interval (CI) 0.83–3.08), but the risk estimates decreased with increasing latency interval. No association was observed during the unexposed period. No increased risk of cardia adenocarcinoma was identified in either period. The risk of non-cardia gastric adenocarcinoma was increased in the potential tamoxifen period (SIR 1.27, 1.03–1.57), and almost doubled (SIR 1.86, 95% CI 1.10–3.14) in the period of longest latency (10–14 years). The corresponding overall SIR was increased in the unexposed group also, but here SIR did not increase with longer latency intervals. An increased risk of tobacco-related tumours, that is, oesophageal squamous-cell carcinoma and lung cancer, was limited to the unexposed cohort, indicating that confounding by smoking might explain the increased SIR during the unexposed period. We concluded that there might be a link between tamoxifen and risk of non-cardia gastric adenocarcinoma.

The male predominance is a striking but as yet unexplained characteristic shared by patients diagnosed with oesophageal and gastric adenocarcinoma. The male to female ratio for oesophageal and cardia adenocarcinoma is about 6–7 to 1, whereas this ratio is about 2–3 to 1 for non-cardia gastric adenocarcinoma (Fuchs and Mayer, 1995; Parkin, 2001). As this pattern is not explained by sex differences in the exposure to known risk factors, sex hormonal influence has been hypothesised (Lagergren and Nyren, 1998; Sipponen and Correa, 2002), but requires further investigation.

Whereas the limited available literature does not support a role of oestrogen in the aetiology of oesophageal adenocarcinoma (Lagergren and Nyren, 1998; Lagergren and Jansson, 2005; Lindblad *et al*, 2006a), data are accumulating in favour of oestrogen influence on the aetiology of gastric adenocarcinoma, a decreased risk of which has been reported among women treated with postmenopausal hormone replacement therapy (HRT) (Lindblad *et al*, 2006a) and among men with prostate cancer treated with oestrogen (Lindblad *et al*, 2004), suggesting that oestrogen has a protective effect.

Tamoxifen is a selective oestrogen receptor modulator with antioestrogenic effects in the breast and oestrogenic activity in various tissues such as the endometrium and bone. It has been widely used in the treatment of oestrogen-dependent breast cancer since the drug was introduced about 30 years ago, and its use in Sweden has been widespread since the late 1980s (Fornander *et al*, 1989). To explore the potential role of sex hormonal influence in the aetiology of oesophageal and gastric adenocarcinoma, we investigated whether tamoxifen-treated postmenopausal women with breast cancer were at increased risk of developing oesophageal or gastric adenocarcinoma in a nationwide Swedish population-based cohort study.

## MATERIALS AND METHODS

### Study cohort

The Swedish Cancer Register was established in 1958 in order to build a complete cancer database for clinical and epidemiological research purposes. All clinicians and pathologists in Sweden are obliged to report all cancer cases to the Registry. Validation studies have shown that the register has a completeness rate of 98% (Lindblad *et al*, 2006b) and virtually all cancer cases (99%) are morphologically verified (Socialstyrelsen, 2005). In addition to the cancer diagnosis, the Cancer Register contains data on sex, date of birth, date of diagnosis, codes for specific sites and histology of cancers, and hospital codes. The Register was used to identify a study cohort of all postmenopausal women (age ⩾50 years) in Sweden with a first primary breast cancer during the period 1 January 1961 to 31 December 2003. For possible confounding by smoking, we also analysed the risk of tobacco-related cancers, that is, oesophageal squamous-cell carcinoma and lung cancer. Only patients with a first and primary breast cancer were included, that is, patients with a previous cancer were excluded, as were patients with a secondary breast cancer. The patients were identified by a unique 10-digit personal registration number given to each Swedish resident at birth or at immigration. This identifier made register linkages possible.

### Follow-up

Crosslinkage within the Cancer Register identified all patients in our cohort who at any time after the first diagnosis of breast cancer developed an oesophageal or gastric adenocarcinoma, with the following ICD codes for the period 1961–1968: oesophageal cancer 150, gastric cancer 151; 1969–1986: oesophageal cancer 150, gastric cancer 151.0/1/8/9; 1987–1996: oesophageal cancer 150.0-5/8/9, gastric cancer 151.0-9 except for 151.7; 1997–2003: oesophageal cancer C15.0-5/8/9, gastric cancer C16.0-9 except for C16.7. The histology code for adenocarcinoma was 096 and for squamous-cell carcinoma 146, and these codes were used during the entire study period. As the Cancer Register has been nationwide since 1961, this was used as the start of the follow-up period for our cohort. This register has distinguished between cardia and non-cardia gastric cancer since 1970. Linkage to the Registers of Causes of Death and Emigration identified deaths and emigrations among the cohort members during the study period.

### Statistical analysis

Relative risks were estimated by calculating the standardised incidence ratio (SIR), that is, the ratio of observed to expected number of newly diagnosed oesophageal and gastric cancer cases. The expected number of cases was calculated by multiplying the observed number of person-years by age-specific and calendar year-specific incidence rates, in 5-year interval, derived from the entire Swedish female population. When calculating the 95% confidence interval (CI) of SIR, we assumed that the number of cases followed a Poisson distribution. All oesophageal and gastric cancer diagnoses occurring within 1 year of follow-up after the primary breast cancer diagnosis were excluded to minimise surveillance bias, that is, a patient with a newly diagnosed breast cancer would be more likely to have another cancer diagnosis detected because of the ongoing diagnostic investigations for the breast cancer. As widespread use of tamoxifen in Sweden only began in the late 1980s (Fornander *et al*, 1989), we considered the cohort of women diagnosed with breast cancer up to the end of December 1987 as being unexposed to tamoxifen, whereas the cohort diagnosed after that date was considered potentially exposed. Women younger than 50 years of age at the time of the breast cancer diagnosis were considered premenopausal and were excluded from the analysis.

### Ethics

The regional ethics committee at Karolinska Institutet in Stockholm, Sweden, approved this study.

## RESULTS

### Oesophageal adenocarcinoma

Among 138 885 postmenopausal women with breast cancer in our total study cohort, contributing with a total of 1 075 724 person-years of follow-up (mean follow-up 7.74 years), we identified 19 patients with oesophageal adenocarcinoma (SIR 1.34, 95% CI 0.85–2.11) (Table 1). In the potentially exposed cohort, nine patients with oesophageal adenocarcinoma were identified, which meant a 60% statistically nonsignificant increase in the risk of developing this tumour (SIR 1.60, 95% CI 0.83–3.08), but the risk estimates decreased with increasing latency interval after breast cancer diagnosis. In the unexposed cohort, the risk of oesophageal adenocarcinoma was not increased (1.17, 95% CI 0.63–2.18), and no trend associated with an increasing latency interval was found (Table 1).

### Gastric cardia adenocarcinoma

Among 119 148 breast cancer patients, contributing 892 555 person-years of follow-up, for whom data on the cardia site were available (from 1970), we identified 21 cases of gastric cardia adenocarcinoma (Table 2). The risk of cardia adenocarcinoma was not increased either in the potentially tamoxifen-exposed cohort (0.96, 95% CI 0.5–1.86) or in the unexposed cohort (SIR 0.75, 95% CI 0.42–1.32), and there was no increase in the risk of cardia adenocarcinoma with increasing latency interval after breast cancer diagnosis (Table 2). The analysis of all gastric cardia cancers (*n*=24), that is, without specification of histologic type, yielded results similar to the analysis of the specified adenocarcinoma cases (data not shown).

### Gastric non-cardia adenocarcinoma

During follow-up of the cohort of 119 148 patients with 892 555 person-years (from 1970), 341 cases of gastric non-cardia adenocarcinoma were identified, which meant an overall increase in the risk of this cancer (SIR 1.41, 95% CI 1.27–1.57) (Table 3). In the potentially tamoxifen-exposed group, there was a 27% statistically significant increase in the risk of gastric non-cardia adenocarcinoma (SIR 1.27, 95% CI 1.03–1.57), and the risk estimates increased with increasing latency time after breast cancer diagnosis. In the longest studied time interval of follow-up in this cohort (10–14 years), the point estimate was increased nearly two-fold (SIR 1.86, 95% CI 1.10–3.14). In the unexposed cohort, an overall 47% statistically significantly increase in the risk was found (SIR 1.47, 95% CI 1.30–1.66), but with increasing latency interval after breast cancer diagnosis, the risk estimates rather decreased (Table 3). In the analyses of all gastric cancers, including tumours without specification of histologic type or gastric subsite (*n*=598), gastric adenocarcinoma without site-specific information (*n*=503), and gastric non-cardia cancer without histologic specification (*n*=405), the risk estimates were all generally similar to those presented for tumours specified as gastric non-cardia adenocarcinomas (data not shown).

### Oesophageal squamous-cell carcinoma and lung cancer

In the cohort unexposed to tamoxifen, there was an overall increase in the risk of oesophageal squamous-cell carcinoma (SIR 1.56, 95% CI 1.21–2.02) and also seemingly in the risk of lung cancer (1.10, 95% CI 0.99–1.22). In the potentially exposed cohort, no such increase in the risk of either oesophageal squamous-cell carcinoma (0.99, 95% CI 0.59–1.64) or lung cancer (0.84, 95% CI 0.73–0.97) was found.

## DISCUSSION

In this study, we found no significant increase in the risk of oesophageal adenocarcinoma among women with breast cancer, and no link between breast cancer and gastric cardia adenocarcinoma. An overall increase in the risk of gastric non-cardia adenocarcinoma was found, and although the risk estimate of gastric non-cardia adenocarcinoma was higher in women not exposed to tamoxifen than in potentially tamoxifen-treated women, an influence of tamoxifen may conceivably still exist. The increased risk of both oesophageal squamous-cell carcinoma and lung cancer during the unexposed period indicates an increased level of smoking among women with breast cancer during this period. No such increases were found during the potential exposure to tamoxifen period. As smoking is linked with a two- to three-fold increase in the risk of gastric non-cardia adenocarcinoma (Lindblad *et al*, 2005), the increased risk in the unexposed cohort might be explained by confounding by smoking, whereas no such confounding could explain the statistically significant increase in risk in the potentially tamoxifen-exposed cohort. Moreover, as a longer latency period between breast cancer and gastric cancer could represent a longer duration of tamoxifen exposure in the potentially exposed cohort, the increased risk with increasing latency time, limited to the potentially exposed period, further argues in favour of a tamoxifen-mediated mechanism.

This is the largest study to investigate oesophageal or gastric adenocarcinoma risk after breast cancer. We used a population-based cohort design and included virtually all postmenopausal female breast cancer patients throughout Sweden diagnosed during a period of more than 40 years. This should have reduced the risk of selection bias and chance errors. Furthermore, the Cancer Register data are of high validity (Lindblad *et al*, 2006b). Another strength of the study is the virtually complete follow-up of all cohort members, which was made possible through crosslinkage of complete population registers, and the personal identification numbers that are allocated to each Swedish resident. Moreover, the homogeneous health-care system in Sweden eliminates the risk of misreporting different regions of the country. An important limitation is our lack of information about the tamoxifen treatment in the individual patients, which introduces exposure misclassification. Given that it was not until the late 1980s that tamoxifen treatment started to be used widely in Sweden (Fornander *et al*, 1989), we decided to regard all postmenopausal women with breast cancer before 1988 as not treated with tamoxifen and patients diagnosed later as potentially so treated. Exposure misclassification should however be non-differential and thus dilute any true associations and would therefore not explain the positive associations found in our study.

Furthermore, confounding might have influenced our result. The increased SIR for gastric cancer in our breast cancer cohort indicates that this cohort is exposed to additional risk factors for gastric cancer compared to the background female population. Our analysis of SIR inherently adjusts for confounding by age and calendar year, and as discussed above, we assessed influence of tobacco smoking through analysis of tobacco-related cancer. Confounding by smoking might be of particular relevance for the interpretation of our results. Knowledge of the hazard of smoking may have had a larger impact on the smoking habits of breast cancer patients compared to the background population and more so during the years 1988–2003 than in the earlier period when the risks related to smoking were less known. We could not adjust for other possible confounding factors, that is, socio-economic status, heredity, diet, body mass, reflux disease, and infection with *H. pylori*, but potential confounding any of these factors does not seem likely to explain the pattern of elevated risks identified in this study. Gastrooesophageal reflux and infection with *Helicobacter pylori* have not been associated with breast cancer, thus eliminating these as confounders. Lastly, despite the large number of person-years followed up in this study, the low incidence of oesophageal and cardia adenocarcinoma among women implied imprecise risk estimates, and although we did not identify an association between tamoxifen and oesophageal adenocarcinoma with statistical significance, the point estimates showed that in view of the risk of a type II error, a true association cannot be ruled out. The results regarding gastric cancer were, however, more robust.

The few previous studies that have addressed sex hormonal influence in the aetiology of oesophageal and cardia adenocarcinoma do not support any such influence, which seems to be in line with our results. No decreased risk of oesophageal adenocarcinoma was observed in a cohort of prostate cancer patients treated with oestrogen (Lagergren and Nyren, 1998), or among postmenopausal women treated with oestrogen (Lindblad *et al*, 2006a). Breastfeeding has been associated with a reduced risk of oesophageal adenocarcinoma (Cheng *et al*, 2000), a finding that does suggest sex hormonal influence, but childbearing has not been found to be linked with a risk of this cancer (Lagergren and Jansson, 2005). Moreover, no association between tamoxifen and risk of oesophageal cancer has been previously observed (Andersson *et al*, 1991; Rutqvist *et al*, 1995; Curtis *et al*, 1996; Matsuyama *et al*, 2000).

Our study indicates that women with breast cancer are at increased risk of gastric adenocarcinoma, and antioestrogen therapy might explain this finding. Recently, a unique global pattern in the male to female ratio of the incidence of gastric adenocarcinoma was reported, indicating a 10–15-year delay in this cancer among female subjects compared to male subjects, possibly owing to the oestrogen exposure (Sipponen and Correa, 2002). We have recently reported that men with oestrogen-treated prostate cancer have a reduced risk of developing gastric adenocarcinoma (Lindblad *et al*, 2004), and in postmenopausal women on HRT, the risk of gastric non-cardia adenocarcinoma seems to be more than 50% lower than in those not receiving such therapy (Lindblad *et al*, 2006a). Some reproductive factors have also been studied in relation to gastric cancer risk. Early menarche, late menopause, and nulliparity seem to increase the risk of breast cancer (Bernstein, 2002). A longer fertility period might reduce the gastric cancer risk (La Vecchia *et al*, 1994; Palli *et al*, 1994; Kaneko *et al*, 2003) and multiparity has been linked with a decreased risk (Kaneko *et al*, 2003). These results have been contradicted, however (Plesko *et al*, 1985; La Vecchia *et al*, 1994; Heuch and Kvale, 2000; Inoue *et al*, 2002). There are some previous results that suggest an increased risk of gastric adenocarcinoma among tamoxifen-treated patients. A pooled analysis of three studies in Scandinavia found a nonsignificantly, but nearly three-fold increased risk of gastric cancer (Rutqvist *et al*, 1995), and correspondingly elevated risks have been found also in other studies (Andersson *et al*, 1991; Curtis *et al*, 1996; Matsuyama *et al*, 2000). Finally, a trend of decreased survival among patients with gastric cancer who had been treated with tamoxifen and in whom the gastric cancer was positive for oestrogen receptors was observed in a randomised trial (Harrison *et al*, 1989). Although, taken together, all these previous studies suggest that tamoxifen might play a role in the aetiology of gastric adenocarcinoma, the association has not been established. Our study adds further support however for a harmful effect of antioestrogen therapy with regard to the risk of developing gastric non-cardia adenocarcinoma.

In conclusion, this study indicates that women who have had a breast cancer diagnosis are at an increased risk of later developing non-cardia gastric adenocarcinoma, possibly as a result of tamoxifen exposure. No such effect was clearly demonstrated for oesophageal or cardia adenocarcinoma. This study should encourage further studies of the influence of sex hormonal influence in the aetiology of these tumours.

## Figures and Tables

**Table 1:** Characteristics of a cohort of postmenopausal Swedish women with a breast cancer diagnosis between 1961 and 2003, and SIR, with 95% CI, of oesophageal adenocarcinoma

	**Oesophageal adenocarcinoma**		
	**Unexposed (1961–1987)**	**Exposed (1988–2003)**	**Total cohort (1961–2003)**
No. of breast cancer cases	73 377	65 508	138 885
No. of person-years at risk	711 837	363 887	1 075 724
Mean years of follow-up	9.70	5.55	7.74
Mean age at entry (range)	68 (50–101)	68 (50–104)	68 (50–104)
			
No. of observed oesophageal adeno-carcinoma cases	10	9	19
No. of expected oesophageal adeno-carcinoma cases	8.49	5.61	14.10
			
SIR (95% CI)	1.17 (0.63–2.18)	1.60 (0.83–3.08)	1.34 (0.85–2.11)
			
**Latency interval after breast cancer diagnosis in years**	**Number of observed cases of oesophageal adenocarcinoma SIR (95% CI)**		
1–4	1	5	6
	0.61 (0.08–4.36)	1.85 (0.77–4.44)	1.38 (0.62–3.08)
5–9	3	3	6
	1.68 (0.54–5.22)	1.44 (0.46–4.47)	1.55 (0.69–3.46)
10–14	3	1	4
	1.67 (0.54–5.20)	1.23 (0.17–8.74)	1.53 (0.57–4.10)
>15	3	0	3
	0.90 (0.29–2.81)		0.90 (0.29–2.80)

CI=confidence interval; SIR=standardised incidence ratio.

Patients diagnosed with breast cancer between 1961 and 1987 were considered unexposed to tamoxifen treatment, whereas those diagnosed between 1988 and 2003 were considered exposed. First year of follow-up was excluded.

**Table 2:** Characteristics of a cohort of postmenopausal Swedish women with a breast cancer diagnosis between 1970 and 2003, and SIR, with 95% CI, of gastric cardia adenocarcinoma

	**Gastric cardia adenocarcinoma**		
	**Unexposed (1970–1987)**	**Exposed (1988–2003)**	**Total cohort (1970–2003)**
No. of breast cancer cases	53 643	65 505	119 148
No. of person-years at risk	528 789	363 765	892 555
Mean years of follow-up	9.85	5.55	7.49
Mean age at entry (range)	69 (50–101)	68 (50–104)	68 (50–104)
			
No. of observed gastric cardia adenocarcinoma cases	12	9	21
No. of expected gastric cardia adeno-carcinoma cases	15.95	9.28	25.24
			
SIR (95% CI)	0.75 (0.42–1.32)	0.96 (0.5–1.86)	0.83 (0.54–1.27)
**Latency interval after breast cancer diagnosis in years**	**Number of observed cases of gastric cardia adenocarcinoma SIR (95% CI)**		
1–4	3	5	8
	0.73 (0.23–2.28)	1.03 (0.43–2.48)	0.89 (0.44–1.79)
5–9	3	3	6
	0.68 (0.21–2.11)	0.93 (0.30–2.89)	0.78 (0.35–1.75)
10–14	3	1	4
	0.85 (0.27–2.65)	0.82 (0.11–5.86)	0.84 (0.31–2.26)
>15	3	0	3
	0.75 (0.24–2.33)		0.74 (0.24–2.32)

CI=confidence interval; SIR=standardised incidence ratio.

Patients diagnosed with breast cancer between 1970 and 1987 were considered unexposed to tamoxifen treatment, whereas those diagnosed between 1988 and 2003 were considered exposed. First year of follow-up was excluded. The earliest data available for the gastric cancer diagnosis were from 1961, but distinction between cardia and non-cardia diagnoses was only available from 1970.

**Table 3:** Characteristics of a cohort of postmenopausal Swedish women with a breast cancer diagnosis between 1970 and 2003, and SIR, with 95% CI, of gastric non-cardia adenocarcinoma

	**Gastric non-cardia adenocarcinoma**		
	**Unexposed (1970–1987)**	**Exposed (1988–2003)**	**Total cohort (1970–2003)**
No. of breast cancer cases	53 643	65 505	119 148
No. of person-years at risk	528 789	363 765	892 555
Mean years of follow-up	9.85	5.55	7.49
Mean age at entry (range)	69 (50–101)	68 (50–104)	68 (50–104)
			
No. of observed gastric non-cardia adenocarcinoma cases	257	84	341
No. of expected gastric non-cardia adeno-carcinoma cases	174.52	65.88	240.39
			
SIR (95% CI)	1.47 (1.30–1.66)	1.27 (1.03–1.57)	1.41 (1.27–1.57)
			
**Latency interval after breast cancer diagnosis in years**	**Number of observed cases of gastric non-cardia adenocarcinoma SIR (95% CI)**		
1–4	105	46	151
	1.7 (1.4–2.06)	1.29 (0.96–1.72)	1.55 (1.32–1.82)
5–9	71	24	95
	1.4 (1.11–1.77)	1.06 (0.71–1.58)	1.3 (1.06–1.58)
10–14	39	14	53
	1.19 (0.87–1.63)	1.86 (1.1–3.14)	1.32 (1.008–1.72)
>15	42	0	42
	1.41 (1.04–1.91)		1.4 (1.04–1.9)

CI=confidence interval; SIR=standardised incidence ratio.

Patients diagnosed between 1970 and 1987 were considered unexposed to tamoxifen treatment, whereas those diagnosed between 1988 and 2003 were considered exposed. First year of follow-up was excluded. The earliest data available for the gastric cancer diagnosis were from 1961, but distinction between cardia and non-cardia diagnoses was only available from 1970.
